# Improvement of Eustachian Tube Function by Tissue-Engineered Regeneration of Mastoid Air Cells

**DOI:** 10.1002/lary.23626

**Published:** 2012-10-19

**Authors:** Shin-ichi Kanemaru, Hiroo Umeda, Masaru Yamashita, Harukazu Hiraumi, Shigeru Hirano, Tatsuo Nakamura, Juichi Ito

**Affiliations:** Department of Otolaryngology, The Foundation for Biomedical Research and InnovationKobe, Japan; the Department of Otolaryngology–Head and Neck Surgery, Medical Research Institute, Kitano HospitalOsaka, Japan; Department of Otolaryngology, Shizuoka General HospitalShizuoka, Japan; Department of Otolaryngology–Head and Neck Surgery, Graduate School of Medicine, Kyoto UniversityKyoto, Japan; Department of Bioartificial Organs, Institute for Frontier Medical Sciences, Kyoto UniversityKyoto, Japan

**Keywords:** Regeneration of mastoid air cells, Eustachian tube function, in situ tissue engineering, middle ear pressure, intractable otitis media

## Abstract

**Objectives/Hypothesis:**

Most cases of chronic otitis media (OMC) are associated with poor development of the mastoid air cells (MACs) and poor Eustachian tube (ET) function. We have previously reported that MAC regeneration can effectively eliminate intractable OMC. In this study, we assessed the ability of regenerated MACs to restore normal gas exchange function and contribute to improved ET function.

**Study Design:**

Clinical trial with control.

**Setting:**

General hospitals.

**Materials and Methods:**

Seventy-six patients with OMC, including cholesteatoma and adhesive otitis media, received tympanoplasty and MAC regeneration therapy. At the first-stage of tympanoplasty, artificial pneumatic bones and/or autologous bone fragments were implanted into the opened mastoid cavity. At the 2nd-stage operation, a nitrous oxide (N2O) gas study was performed in 10 patients to measure middle ear pressure (MEP). For the control group, MEP was measured in five patients with good MAC development during cochlear implantation or facial nerve decompression. ET function was measured twice in each patient, once before the 1st operation and 6 months after the second operation.

**Results:**

At the 2nd-stage operation, in all cases with regenerated MACs and in the normal control group, MEP changed after administration of N2O. In contrast, no change in MEP was observed in cases with unregenerated MACs. In 70% (n = 37/53) of the regenerated MAC group, ET function was improved, whereas improvement of ET function was observed in only 13% (n = 3/23) of the unregenerated MAC group.

**Conclusions:**

Tissue-engineered regeneration of MACs improves ET function and gas exchange in the middle ear. Laryngoscope, 2012

**Level of Evidence:**

3b

## INTRODUCTION

Most chronic otitis media (OMC), including cholesteatoma and adhesive otitis media, are associated with poor development of the mastoid air cells (MACs) and poor Eustachian tube (ET) function. This means that disorder of gas exchange functions of middle ear may be one of the major causes of OMC.

A common treatment for OMC is tympanoplasty with mastoidectomy, the purpose of which is lesion removal and reconstruction of the sound conduction system. However, tympanoplasty with mastoidectomy does not directly aim for the recovery of MACs and ET function. While in some cases removal of the lesions can induce functional recovery, cholesteatoma and adhesive otitis media will often recur to some degree during long-term observation. The fundamental problems of OMC are clearly not resolved through ordinary tympanoplasty with mastoidectomy. In order to achieve complete recovery from intractable otitis media, it is necessary to regenerate MACs and ET function.

We have previously reported that regeneration of MACs can effectively eliminate intractable OMC.^1^–^3^ In this study, we assessed the ability of regenerated MACs to restore gas exchange function and contribute to the improvement of ET function.

This is the first clinical report that investigated the relationships between the regenerated MACs and ET function.

## MATERIALS AND METHODS

### Patients

TABLE I shows the patient profile. Eighty-one patients participated in this study. Seventy-six patients with cholesteatoma, adhesive otitis media, or chronic otitis media received tympanoplasty with mastoidectomy and MAC regeneration therapy in a two-stage operation. During the first-stage operation, artificial pneumatic bones (n = 19) and/or autologous bone fragments (n = 57) were implanted into the opened mastoid cavity. During the 2nd-stage operation, a nitrous oxide (N2O) gas study was performed in five patients with good MAC regeneration and five patients with poor MAC regeneration to measure middle ear pressure (MEP) through the opened bar hole of the mastoid. For the control group, MEP was measured in five patients with well-developed MACs during cochlear implantation (n = 3) or facial nerve decompression (n = 2). ET function was also measured twice in each patient, once before the first-stage operation and 6 months after the second-stage operation.

**TABLE I:** 

N = 81, M/F: 35/46, Age: 2–83 (Avg. 52.3)		
Tympanoplasty and MAC regeneration group (Poor development of mastoid air cells)		n = 76
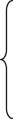	Simple chronic otitis media	n = 24
	Adhesive otitis media (AOM)	n = 6
	Cholesteatoma (including cholesteatoma with AOM)	n = 46
Control group (Good development of mastoid air cells)		n = 5
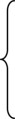	Facial nerve palsy (Facial nerve decompression)	n = 2
	Profound hearing loss (Cochlear implantation)	n = 3

### Regenerative Operation

**Fig. 1:**
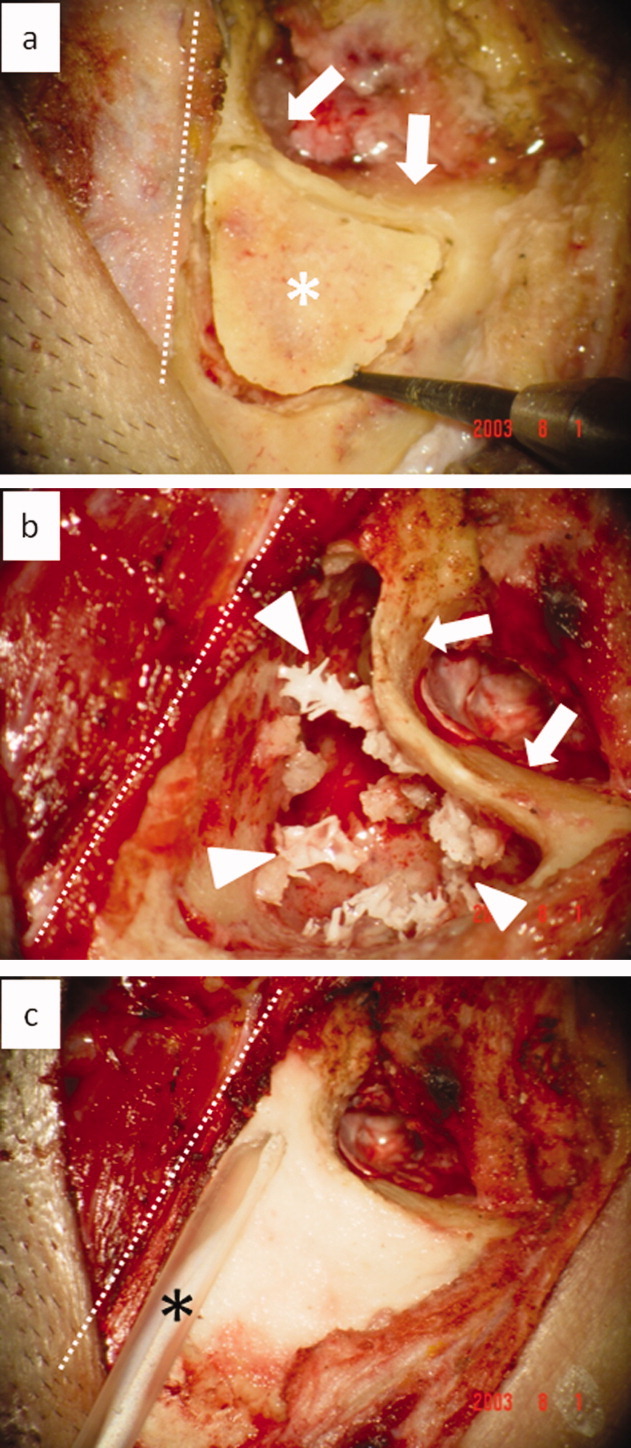
Regenerative first-stage operation with mastoid cortex plasty (right ear). white dotted line, temporal line; white asterisk, mastoid cortex bone lid; white arrow, posterior wall of external auditory meatus; white arrow head, artificial pneumatic bones; black asterisk, drainage tube. a. Before the mastoidectomy is performed, a groove is made on the surface of the post-auricular cortex bone using a minimum-size cutting bar. Bone powder, for making bone putty, is taken from outside the groove. Mastoid cortical bony plate was quarried out from the surface of the temporal bone behind the external auditory canal before mastoidectomy to cover the opened mastoid cavity at the end of the operation. b. After the cortex lid is chiseled, mastoidectomy is performed in the usual manner. Cholesteatoma, granulation and other lesions are removed while preserving the healthy mucosa as much as possible in this region. Collagen-coated hydroxyapatite fragments (artificial pneumatic bones) are transplanted sparsely into the opened mastoid cavity and fixed in place by fibrin glue. c. Bone putty mixed with fibrin glue is applied to the edge of the opened mastoid cavity, then the cortex lid is returned to its original position. The cortex lid is then fixed and covered with bone putty. The drainage tube is placed into the mastoid cavity, and the surface of this region is made smooth using the finger cushion. Finally, fibrin glue is sprinkled over this region. [Color figure can be viewed in the online issue, which is available at wileyonlinelibrary.com.]

The operative method for MAC regeneration was performed as described in our previous reports.^1^–^3^ In this study, we used artificial pneumatic bones covered with atelocollagen and/or autologous bone fragments that were harvested during mastoidectomy as base materials for regenerating the MACs. Figure 1 shows the operative procedures for the first regenerative operation, which includes implantation of artificial pneumatic bones into the newly opened mastoid cavity and cortex plasty.

Assessment of recovery of mastoid aeration and regeneration of the pneumatic air cells of the mastoid cavity were performed by high resolution computed tomography (HRCT) scan images taken before and then 6 months after the first- and second-stage operations, respectively. There are many reports in the literature regarding the usefulness of HRCT scan images in the evaluation of the MAC system.^4^

Assessment of good regeneration of mastoid air cells were determined when both aeration up to the mastoid antrum and new bony trabeculation (pneumatic air cells) of mastoid cavity were identified by the images of HRCT.

### Measurement of MEP

**Fig. 2:**
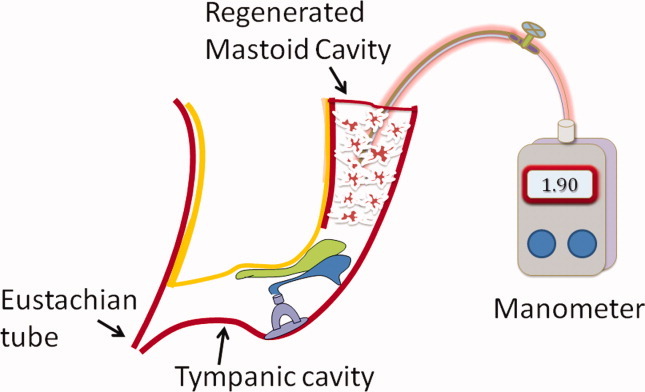
Measurement of middle ear pressure. [Color figure can be viewed in the online issue, which is available at wileyonlinelibrary.com.]

Between 8 and 14 months after the first-stage operation, the second-stage operation was performed. Just before the second-stage operation, we checked for the presence of good aeration in the newly regenerated MACs using HRCT scan images. Five patients were selected, each with either good or no aeration in the newly opened mastoid cavity. At the second-stage operation, the bar hole was opened in the regenerated mastoid cortex bone and a 2-mm diameter elastic tube was inserted into the hole. After sealing the bar hole with bone wax, the elastic tube was connected to a micropressure sensor (Handheld Digital Manometer: Copal Electronics, Tokyo, Japan; Fig. 2). Under general anesthesia with sevoflurane, changes in MEP were measured with the micropressure sensor every 1 minute during the 20 minutes after administration of N2O gas in each of the 10 patients. MEP was measured the same way in the five patients of the control group with good MAC development during cochlear implantation (n = 3) or facial nerve decompression (n = 2).

### Measurement of ET function

ET ventilation function was measured by sonotubometry using an instrument designed to assess ET function (JK-05A: RION Co., Ltd, Tokyo, Japan). Measurements were taken twice in each patient, once before the first-stage operation and again 6 months after the second operation.

## RESULTS

### Regeneration of MACs

**Fig. 3:**
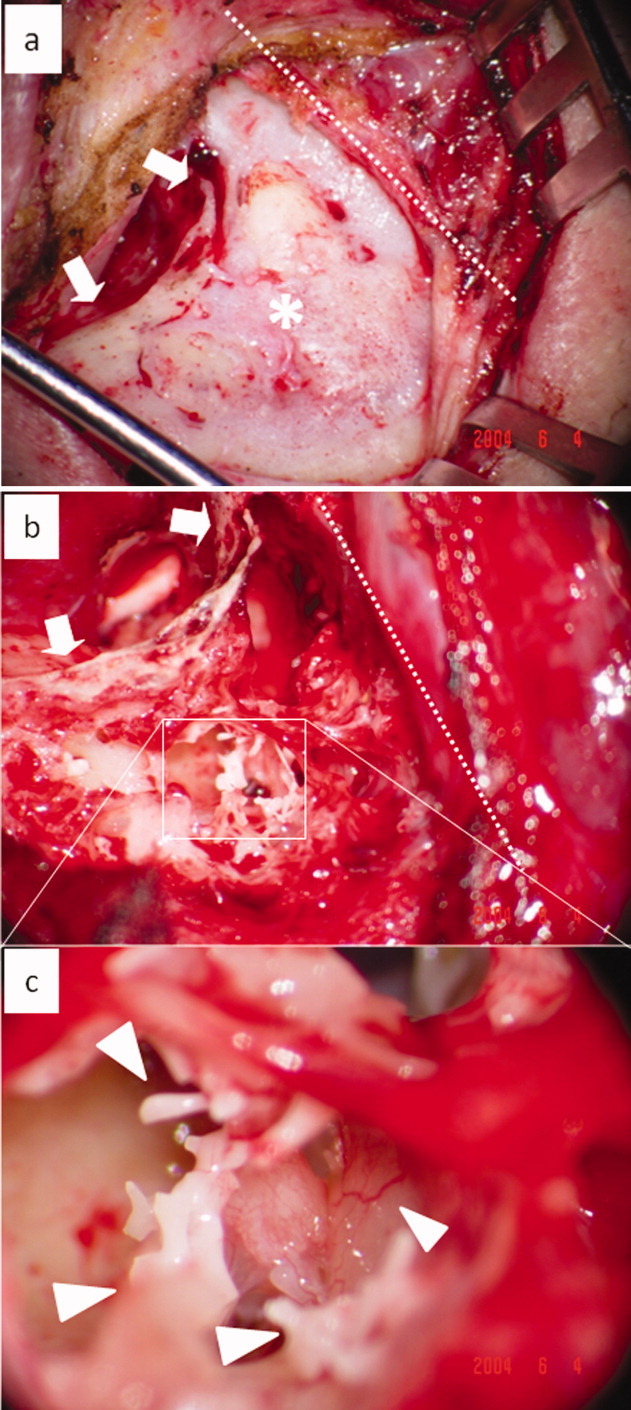
Regenerated mastoid air cells after reopening the mastoid cavity at the second-stage operation (left ear) (white dotted line, temporal line; white asterisk, regenerated mastoid cortex bone; white arrow, posterior wall of external auditory meatus; white arrow head, artificial pneumatic bones). (a.) The mastoid cortex bone showed complete regeneration 1 year after the first-stage operation. (b.) Regenerated mastoid air cells and good aeration were observed after reopening the mastoid cavity. (c.) Enlargement of regenerated mastoid air cells. [Color figure can be viewed in the online issue, which is available at wileyonlinelibrary.com.]

Good MAC regeneration was observed in 26.3% (20/76) of the cases before the second-stage operation. Six months after the second-stage operation, the success rate of MAC regeneration had increased to 69.7% (53/76). Figure 3 shows a representative case of good MAC regeneration at the second-stage operation.

### Changes in MEP

**Fig. 4:**
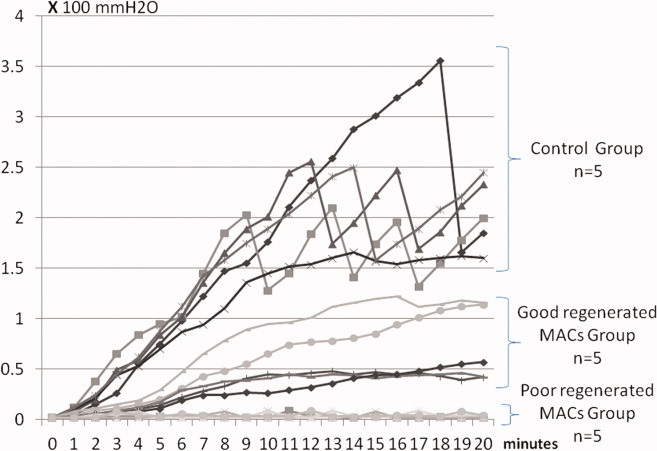
Changes in middle ear pressure after administration of N2O gas. [Color figure can be viewed in the online issue, which is available at wileyonlinelibrary.com.]

Figure 4 shows the changes in MEP within the three groups. In the control group, MEP increased rapidly and reached over 150mm H2O after administration of N2O gas in all cases. MEP in the good MAC regeneration group was also increased after administration of N2O gas in all cases; however, both maximum MEP and the increasing ratios were lower than those of the control group. In contrast, no changes in MEP were observed in the poor MAC regeneration group throughout the measurement period.

### The Relationship Between MAC Regeneration and ET Function

Prior to operation, good ET function was observed in 19.7% (15/76) of the staged operation group and in 100% (5/5) of the control group. Most cases showing good ET function in the staged operation group were OMC patients with tympanic membrane perforation.

**Fig. 5:**
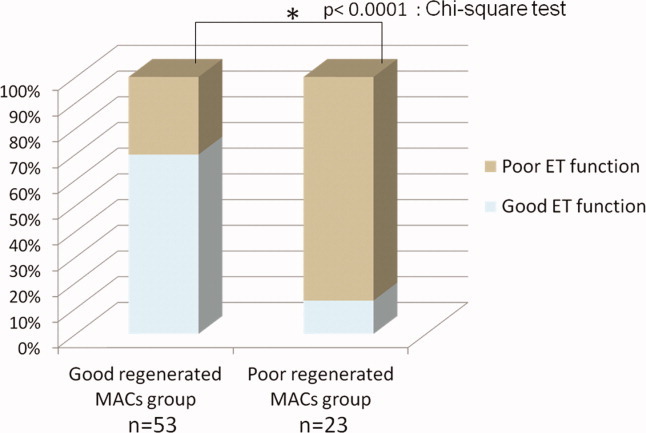
Relationship between MAC regeneration and ET function. [Color figure can be viewed in the online issue, which is available at wileyonlinelibrary.com.]

Six months after the second operation, 52.6% (40/76) of the patients showed improved ET function. Of the 53 cases of good MAC regeneration, 69.8% (37/53) of the cases showed improved ET function. Of the 23 cases of poor MAC regeneration, 13% (3/23) of the cases showed improved ET function. The differences between these two groups were statistically significant (Chi-square test: p<0.0001; Fig. 5).

In both groups of good and poor MAC regeneration in Figure 4, the average opening times of ET while swallowing 2 or 3 times, both before and 6 months after the second operation, are indicated in Table II. This table shows that regeneration of MACs correlates closely with recovery of ET function and gas exchange function.

**TABLE II:** 

Group	Before op Avg. duration (ms)	After op Avg. duration (ms)
	—	257
Good regenerated	83	112
MACs group	—	68
	—	—
	—	181

	—	—
	—	—
Poor regenerated	*103	—
MACs group	—	—
	—	—

Avg. duration indicates average opening time of ET during 2 or 3 times swallowing.

Minus (−) indicates under 50 ms of opening time.

* Simple OMC with TM perforation

## DISCUSSION

Tympanoplasty with mastoidectomy is presently the best operative treatment for OMC, including cholesteatoma, adhesive otitis media, and others. However, recurrence of OMC is common even after operation. Most cases of OMC show poor development of MACs and poor ET function. MEP is regulated not only by the ET but also by MACs. The ET plays the leading role in MEP regulation through swallowing.^5^ MACs are covered by a mucosa that contains numerous capillaries through which gas exchange is performed depending on MEP.^6^–^10^ Thus, the ET plays a rapid and active role in MEP regulation, whereas MACs play a slow and passive role in MEP regulation.^9^

The maintenance of MEP at atmospheric levels, which is required for good sound conduction, is one of the most important functions of the MAC system. Once this function fails, otitis media follows a protracted course.^9^, ^10^

Our previous study showed that mastoid air cells could be regenerated by implantation of artificial pneumatic bones.^1^–^3^

In this study, we observed that MEP increased in the group with good MAC regeneration after administration of N2O gas. This indicates that regenerated MACs can perform gas exchange functions, although they do not reach the levels obtained by normal well developed MACs. The gas exchange function of MACs is thought to be proportionate to the amount of capillary-containing mucosa spread over their surface. Thus, in order to improve gas exchange function, it is essential to generate space to increase the surface area of mucosa-covered MACs. Traditional mastoidectomy is not sufficient to create enough space to increase gas exchange function. In an attempt to increase the surface area available for mucosal growth, we implanted artificial pneumatic bones coated with atelocollagen and/or autologous bone fragments into the newly opened mastoid cavity and performed mastoid cortex plasty.

**Fig. 6:**
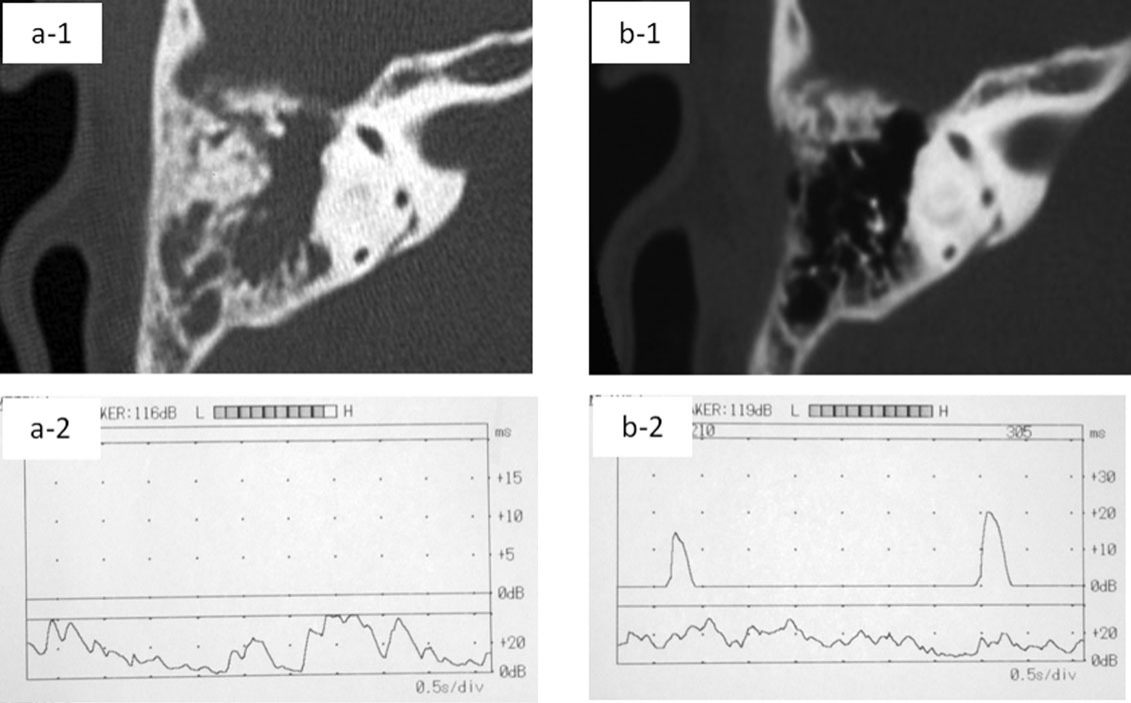
Typical case of improved Eustachian tube function after MAC regeneration. Nine-year-old female with cholesteatoma with adhesive otitis media. (a-1, b-1) High-resolution CT scan of the temporal bone. (a-2, b-2) Sonotubometry test results for Eustachian tube ventilatory function. (a) Pre-operation. Development of mastoid air cells and Eustachian tube function is poor and there is no aeration in the mastoid cavity. (b) Six months after the second-stage operation. Regenerated mastoid air cells and good aeration in the mastoid cavity are observed along with concurrent improvement of Eustachian tube function.

This study found significant improvement in ET function after operation in many cases of good MAC regeneration (Fig. 5,6); however, improvement was not observed in cases of poor MAC regeneration. This demonstrates that recovery of MAC gas exchange function also improves ET function. We think the mechanism of this result as follows: negative pressure in the middle ear cavity caused by disorder of gas exchange function locks on opening of ET. Gas enters into mastoid cavity through capillaries on regenerated MACs. This releases negative pressure in the middle ear cavity. This makes it easy to open ET. Thereafter, it contributes to normalize gas exchange function in the middle ear.

It is important to note that of the cases with good ET function prior to operation, 80.0% (12/15) of the cases were simple OMC with tympanic membrane perforation. It is thought that ET function was not accurately reflected in these cases because there was no pressure difference between the internal and external portions of the middle ear cavity. It is therefore possible that improvement in ET function occurs after operation in a greater number of cases with regenerated MACs. Clinically, we have observed few cases of poor ET function with good development of MACs, and vice versa. This suggests that a mutualistic relationship exists between MAC function and the ET.

We found promising indications that intractable OMC may be effectively treated using tissue engineering methods of MAC regeneration. However, given that our success rate of MAC regeneration is under 70%, and gas exchange function is low compared with normally functioning MACs, further study will be needed to achieve a higher rate of recovery.

## CONCLUSION

This study shows that:

1. Regenerated MACs can perform gas exchange function in the middle ear, though their function cannot reach the level of normally developed MACs.
2. ET function improved after operation in many cases of MAC regeneration.
3. There are considered to be the mutual-comprehensive relationships between the function of MACs and ET.
